# Synthesis and Characterization of Magnetoelectric Ba_7_Mn_4_O_15_

**DOI:** 10.1021/acs.inorgchem.2c00889

**Published:** 2022-06-21

**Authors:** Gabriel R. M. Clarke, Martin R. Lees, Clemens Ritter, Ivan da Silva, Mark S. Senn

**Affiliations:** † Department of Chemistry, University of Warwick, Coventry CV4 7AL, U.K.; ‡ Department of Physics, University of Warwick, Coventry CV4 7AL, U.K.; § Institut Laue-Langevin, 38042 Grenoble Cedex 9, France; ∥ ISIS Neutron and Muon Facility, Rutherford Appleton Laboratory, Didcot OX11 0QX, U.K.

## Abstract

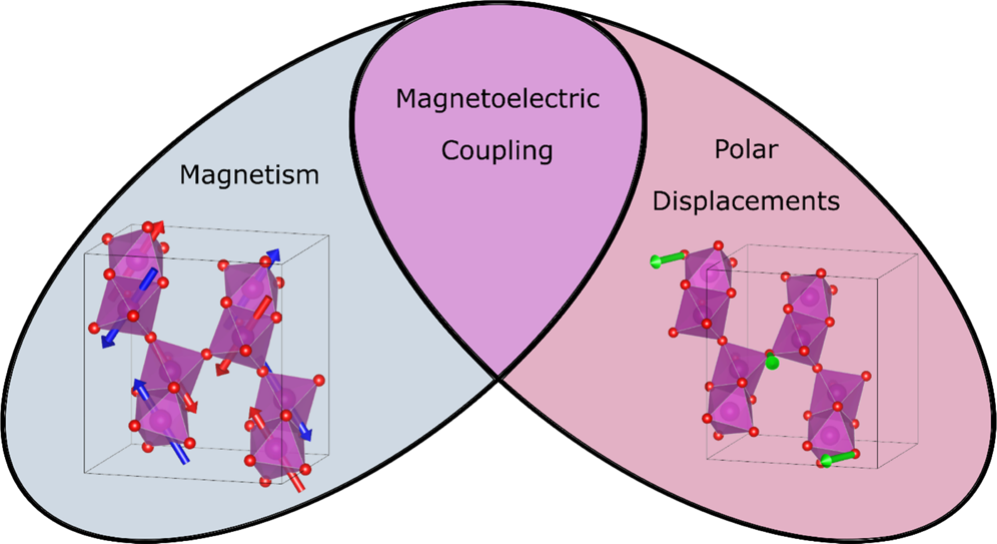

We present the synthesis of a novel binary metal oxide material: Ba_7_Mn_4_O_15_. The crystal structure has been investigated by high-resolution powder synchrotron X-ray diffraction in the temperature range of 100–300 K as well as by powder neutron diffraction at 10 and 80 K. This material represents an isostructural barium-substituted analogue of the layered material Sr_7_Mn_4_O_15_ that forms its own structural class. However, we find that Ba_7_Mn_4_O_15_ adopts a distinct magnetic ordering, resulting in a magnetoelectric ground state below 50 K. The likely magnetoelectric coupling mechanisms have been inferred from performing a careful symmetry-adapted refinement against the powder neutron diffraction experiments, as well as by making a comparison with the nonmagnetoelectric ground state of Sr_7_Mn_4_O_15_.

## Abstract 2

The synthesis, structural characterization, and magnetic analysis of a novel solid-state phase, Ba_7_Mn_4_O_15_, are presented. While the high-temperature phase was found to be isostructural to Sr_7_Mn_4_O_15_, a detailed symmetry-based analysis of low-temperature neutron powder diffraction data, combined with magnetic susceptibility measurements, reveals that Ba_7_Mn_4_O_15_ has a multiferroic ground state.

## Introduction

Solid-state phases containing Mn–O systems are of great interest due to the variety of functional properties they may exhibit, such as colossal magnetoresistance, ferromagnetism (FM), and ferroelectricity (FE).^1^ Multiferroic materials combine the latter two properties in a single material and have gained popularity recently for both their fundamental chemical novelty and their potential applications in devices.^2^ If the onset of magnetic and ferroelectric orderings have different mechanisms, the phase is known as a type I multiferroic. These phases typically have different FM and FE ordering temperatures, and the coupling between the two properties is weak. In a type II multiferroic, the magnetic ordering is induced by the ferroelectric ordering or vice versa, leading to a single ordering temperature and strong coupling between the ferroic states. One of the best-known examples of a Mn–O-containing multiferroic is YMn_2_O_5_, an orthorhombic *Pbam* structure comprising edge-shared chains of Mn^IV^O_6_ octahedra along the **c** axis that are linked by Mn^III^O_5_ pyramids.^3^ The mechanism of the multiferroicity in this phase has been identified as exchange striction, in which the onset of magnetic ordering causes a polar structural distortion.^4,5^ All of the resulting components of the electrical polarization lie along the **b** axis.^6^ Another example of a Mn-containing multiferroic is TbMnO_3_, in which the FE polarization is moderated by the Dzyaloshinkii–Moriya (DI) interaction and is thus quite weak.^7−9^ The arrangement of the Mn–O polyhedra in these phases has significant effects on the magnetoelectric coupling, and other multiferroics may be discovered by exploring phases containing unusual Mn–O linkages. A variety of type II multiferroic Mn–O-containing phases may already be found in the literature with ferroelectricity, which is induced by improper mechanisms, magnetic ordering, charge ordering, or orbital ordering.^10−15^

The Sr_7_Mn_4_O_15_ phase was first described by Kriegel et al.; it crystallizes in the *P*2_1_/*c* space group and contains face-sharing Mn_2_O_9_ octahedral dimers, which share corners to form strings in the **ac** plane; these strings stack along **a** to give a quasi-two-dimensional structure.^16,17^ The face-sharing dimer motif is uncommon compared to solely corner-sharing systems, appearing in 4H-SrMnO_3_ and as infinite chains in 2H-BaMnO_3_, and in perovskites has been shown to appear as a particular result of relative ionic sizes.^18^ In Sr_7_Mn_4_O_15_, these dimer units result in strong antiferromagnetic (AFM) interactions between the neighboring Mn^4+^ sites, a broad peak in the DC magnetic susceptibility and divergence between the field-cooled cooling (FC) and zero-field-cooled (ZFC) warming curves.^19,20^ In addition to the broad maximum, we have previously observed divergences between the FC and ZFC susceptibility results for the series Sr_7_Mn_4_O_15_ to Sr_3.5_Ca_3.5_Mn_4_O_15_ below 175 K, leading us to suggest that the behavior could be explained by weak ferromagnetism (wFM) in which the AFM spins are slightly canted. We propose that the space group in which this is allowed, *P*2_1_, would also allow local displacements of the oxide anions to produce FE ordering, resulting in a magnetoelectric ground state for the phase.^21^ In general, the low symmetry of this structural type means that a large number of ways exist in which the magnetic exchange interactions may break inversion symmetry and hence allow for the magnetoelectric effect. Motived by this fact and previous reports of limited isovalent substitutions on the Sr site, we have prepared the Ba analogue Ba_7_Mn_4_O_15_, which represents a novel binary oxide of Ba and Mn. Our detailed characterization of the magnetic ground state suggests that it may possess magnetoelectric coupling.

## Experimental Details

To synthesize Ba_7_Mn_4_O_15_, BaCO_3_ (99.95%, Alfa Aesar) and MnO_2_ (99.996%, Alfa Aesar) were ground together in a 7.7:4 ratio (i.e., a 10% molar excess of BaCO_3_ compared to a stoichiometric reaction) and pressed into a pellet (diameter: 13 mm) under 7.5 metric tonnes of force. The pellet was heated to 900 °C for 48 h, then reground, pressed, and heated to 900 °C again. The grinding, pressing, and heating process was performed five times, and heating was always performed under an atmosphere of flowing N_2_.

To synthesize Sr_7_Mn_4_O_15_, SrCO_3_ (99.9%, Sigma-Aldrich) and MnO_2_ (99.996%, Alfa Aesar) were ground together in a 7:4 ratio and pressed into a pellet (diameter: 13 mm) under 7.5 metric tonnes of force. The pellet was heated to 900 °C for 20 h, then reground, pressed, and heated to 1000 °C for 24 h. The grinding, pressing, and heating process was performed six times under air.

High-resolution powder synchrotron X-ray diffraction experiments were performed at Beamline I11 at Diamond Light Source, with diffraction patterns recorded at 300 and 100 K using the multianalyzer crystal (MAC) for both Ba_7_Mn_4_O_15_ and Sr_7_Mn_4_O_15_, and variable-temperature measurements performed between these temperatures using the Mythen detector for Ba_7_Mn_4_O_15_. The beam wavelength was 0.826831 Å for the Ba_7_Mn_4_O_15_ diffraction experiment and 0.826341 Å for the Sr_7_Mn_4_O_15_, refined using NIST 640c Si standards. Powder neutron diffraction experiments were performed using the GEM instrument at the ISIS Neutron and Muon Source at 80 and 10 K for Ba_7_Mn_4_O_15_ and using the D2B instrument at the Institut Laue-Langevin (ILL) at 300, 100, and 1.5 K for Sr_7_Mn_4_O_15_.^22,23^

Rietveld refinements were performed using TOPAS Academic v6.^24^ A starting model for the refinement was based on the crystal structure of the related Sr_7_Mn_4_O_15_ phase with space group **P**2_1_/**c**. Combined Rietveld refinements were performed using the 100 K dataset from the I11 MAC detector and the 80 K dataset from the GEM experiment. These refinements excluded detector bank 1 and bank 6 from the GEM dataset due to poor signal-to-noise ratios. Refined lattice parameters and atomic coordinates for Ba_7_Mn_4_O_15_ at 300 K are summarized in the Supporting Information (Table S1) isotropic displacement parameters were constrained to be equal for a given atom. Refinements of the low-temperature magnetic structure were performed within the symmetry-adapted formalism of the ISODISTORT suite^25^ and as implemented through the linear constraints language of TOPAS. Refined values from these refinements are also summarized in the Supporting Information (Table S2).

Magnetization measurements were performed using a Quantum Design MPMS SQuID magnetometer. Magnetic susceptibility versus temperature data were collected between 10 and 325 K in an applied field of 100 Oe under zero-field-cooled warming and field-cooled cooling conditions. Magnetization versus field curves were collected in applied fields of up to 50 kOe at temperatures between 2 and 300 K.

## Results and Discussion

**Figure 1:**
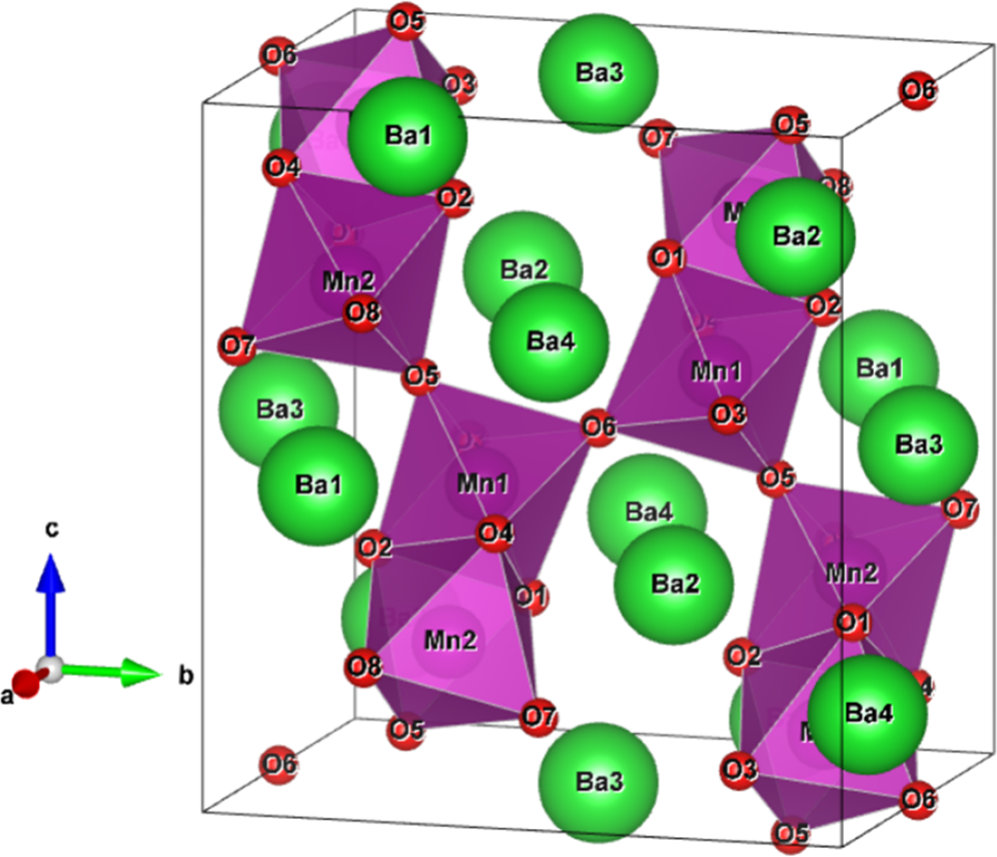
Unit cell of Ba_7_Mn_4_O_15_ with sites labeled. Purple polyhedra represent Mn_2_O_9_ octahedral dimers, green spheres indicate Ba atoms, and red spheres represent O atoms.

Sr_7_Mn_4_O_15_ has been demonstrated to be flexible to limited substitution by both Ca^2+^ and Ba^2+^ cations at the Sr^2+^ sites.^19,20^ Each substituting cation preferentially occupies different Sr sites within the unit cell, with the smaller Ca^2+^ occupying the Sr1 site and the larger Ba^2+^ occupying the Sr3 and Sr4 sites (see Figure 1). However, substitution levels greater than *x* = 1 for Sr_7–*x*_Ba*_x_*Mn_4_O_15_ have not previously been reported. We found that the use of an inert atmosphere and a 10% molar excess of BaCO_3_ in the synthesis of Ba_7_Mn_4_O_15_ was necessary for obtaining the desired phase. Any attempt to synthesize the product in air results in oxidation of the Mn^4+^ cation to Mn^5+^, forming a product which was identified as the Ba_3_Mn_2_O_8_ phase exclusively.^27^ Additionally, performing the reaction at a reduced temperature of 900 °C (compared to literature at 1300 °C) produced the purest material. If an inert atmosphere is used without excess BaCO_3_ in the reaction mixture, the reaction produces a mixture of Ba_7_Mn_4_O_15_ and a secondary phase which was identified as Ba_4_Mn_3_O_10_.^28^ Since Ba_7_Mn_4_O_15_ possesses a greater Ba:Mn ratio than Ba_4_Mn_3_O_10_, we found that a 10% excess of BaCO_3_ produces a near phase-pure product, with just a small quantity of unreacted poorly crystalline BaCO_3_ in the diffraction pattern. The inert atmosphere, requisite excess of BaCO_3_ and exceptionally low synthesis temperature required to stabilize this phase are no doubt contributing factors as to why Ba_7_Mn_4_O_15_ has hitherto remained unidentified.

**Figure 2:**
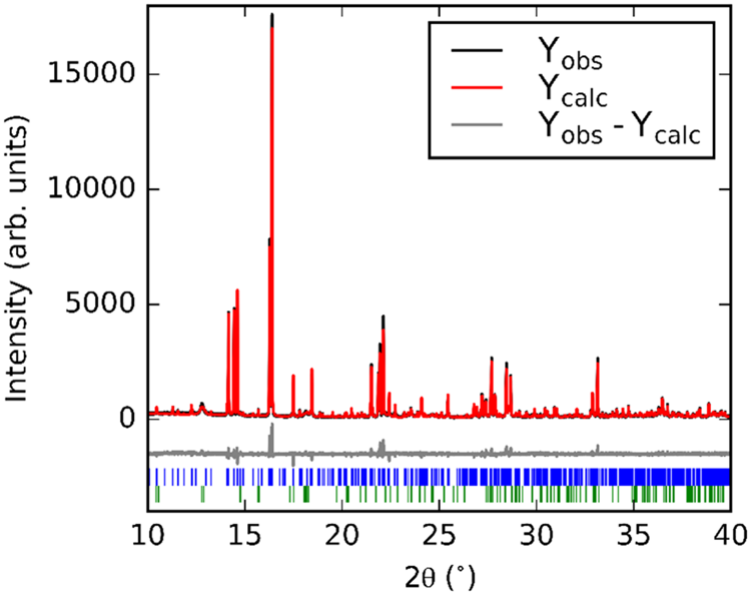
Rietveld refinement against powder synchrotron X-ray diffraction (λ = 0.826831 Å) data at 300 K. Blue tick marks indicate reflections for Ba_7_Mn_4_O_15_; green tick marks indicate reflections for BaCO_3_.

Figure 2 shows the result of a Rietveld refinement against the high-resolution powder synchrotron X-ray diffraction data collected at 300 K of Ba_7_Mn_4_O_15_; the unreacted BaCO_3_ appears as a broad peak around 2θ = 13° with a calculated concentration of ∼12% by weight. This value may be overestimated due to the low crystallinity of the residual reagent. The unit cell of Ba_7_Mn_4_O_15_ is similar to that reported for Sr_7_Mn_4_O_15_, containing the same Mn_2_O_9_ dimer units, but with larger lattice parameters resulting from the greater ionic radius of Ba^2+^ compared to Sr^2+^. At 300 K, we do not find evidence for the disordering of the Sr(3) and O(6) sites from their high-symmetry positions as described by Vente et al. for Sr_7_Mn_4_O_15_.^20^

**Figure 3:**
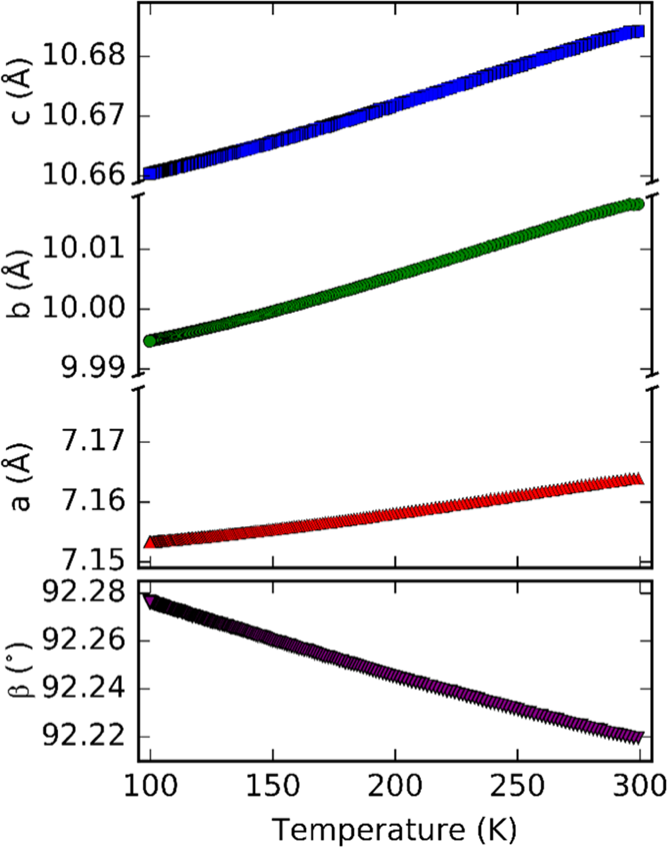
Lattice parameters of Ba_7_Mn_4_O_15_ as a function of temperature.

The variations in lattice parameters and the angle β with temperature between 300 and 100 K are shown in Figure 3. All parameters vary monotonically with temperature, indicating the absence of any structural phase transitions in this temperature range.

In the case of Sr_7_Mn_4_O_15_, the strings of Mn_2_O_9_ dimers were predicted to result in strong magnetic exchange interactions in the **bc** plane, but weak interactions along **a**.^20^ Previous reports of magnetic susceptibility experiments on Sr_7_Mn_4_O_15_ describe a broad maximum centered around 75–90 K^20,21^ with a small upward tail below around 20 K. The FC and ZFC curves diverge from one another at temperatures below this maximum. This behavior has been explained with two different mechanisms: Vente et al. proposed that it resulted from spin glass-like behavior producing clusters of antiferromagnetically ordered spins, which crystallize into true antiferromagnetic order below ∼75 K, whereas we have previously suggested that it might represent a weak FM ordering component, arising from the local symmetry-breaking associated with the aforementioned disorder of the Sr and O sites.^20,21^

**Figure 4:**
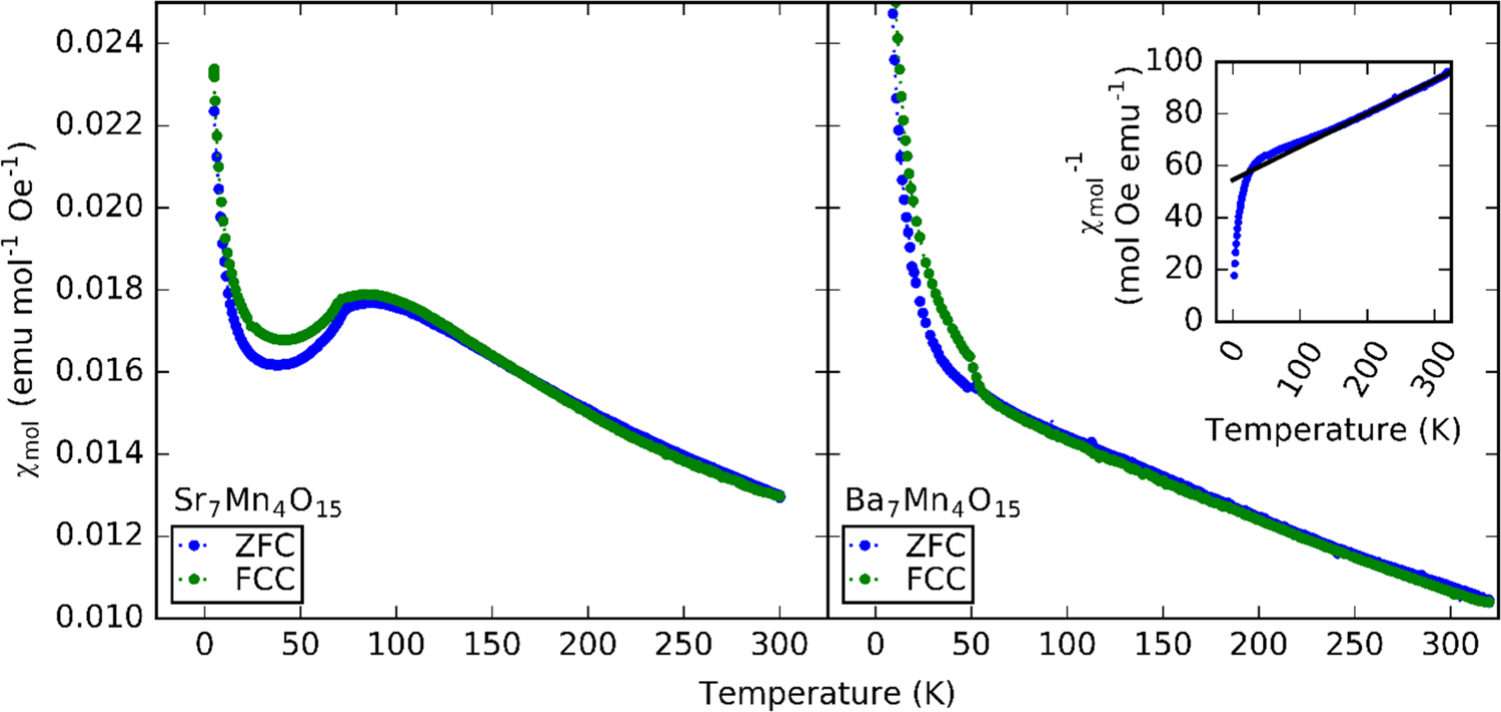
DC susceptibility versus temperature for Sr_7_Mn_4_O_15_ and Ba_7_Mn_4_O_15_. Inset: Curie–Weiss fit between 200 and 300 K for Ba_7_Mn_4_O_15_.

We find that the DC susceptibility versus temperature results for Sr_7_Mn_4_O_15_ match well with previous descriptions, with the maximum of the broad feature centered around ∼84 K and the deviation between the ZFC warming and FC curves below this temperature (Figure 4). In comparison, the DC magnetic susceptibility results for Ba_7_Mn_4_O_15_ are relatively featureless. We observe a steady upward trend with decreasing temperature between 300 and 50 K. However, on further cooling a clear divergence between the FC and ZFC warming curves is visible, evidencing a possible long-range magnetic ordering transition. The susceptibility obeys the Curie–Weiss law in the 200–300 K range. A fit to this part of the inverse susceptibility in the ZFC warming data yields a μ_eff_ = 3.78(7) μ_B_ per Mn^4+^ site for Ba_7_Mn_4_O_15_ that compares favorably against the expected spin-only value of 3.87 μ_B_.

**Figure 5:**
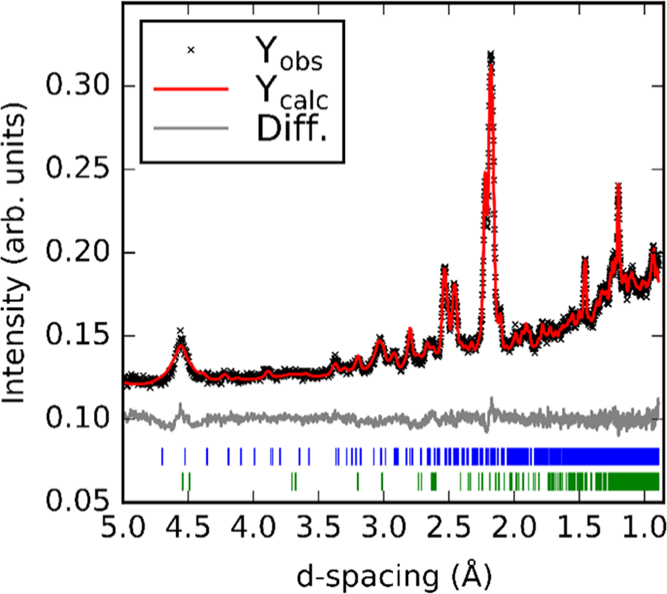
Results of combined Rietveld refinement against GEM and I11 diffraction data at 80 and 100 K, respectively, for Ba_7_Mn_4_O_15_. Visualized is the fit against the data on bank 3 of GEM, though data from all banks was used to generate the model. Blue tick marks indicate reflections for Ba_7_Mn_4_O_15_; green tick marks indicate reflections for BaCO_3_.

**Figure 6:**
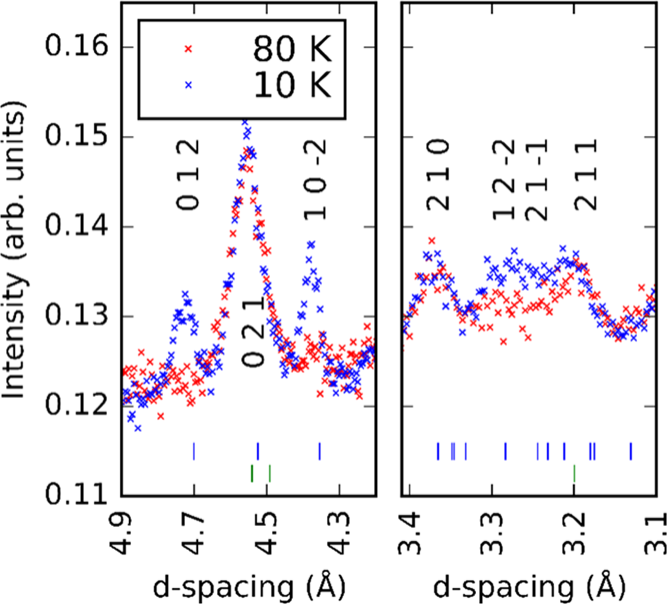
Comparison of the powder neutron diffraction data for Ba_7_Mn_4_O_15_ from GEM bank 3 at 80 and 10 K, showing the development of magnetic Bragg peaks.

To further investigate the magnetic behavior of Ba_7_Mn_4_O_15_, we performed powder neutron diffraction at 10 and 80 K (i.e., either side of the divergence) using the time-of-flight powder diffractometer GEM, ISIS. Figure 5 shows the result of a combined Rietveld refinement using the powder synchrotron X-ray diffraction data at 100 K and the powder neutron diffraction data at GEM at 80 K (the same temperature points not having been measured). A fit to data from the same bank at 10 K is also included in the Supporting Information (Figure S1). This refinement of the neutron diffraction data at 80 K is well above the suspected magnetic ordering temperature and shows no significant unfit intensity by our nuclear model. Figure 6 shows an enhanced view of the *d* = 3.1–3.4 Å and *d* = 4.2–4.9 Å regions of neutron diffraction data from the same bank at 10 K; magnetic Bragg reflections are evident that index as (0 1 2), (1 0 −2), (1 2 −2), and (2 1 −1).

To fit these magnetic peaks and solve the magnetic structure, the nuclear structure refined using the combined 80 and 100 K data above was used to produce a .cif file, which was used as a starting model in ISODISTORT for the 10 K neutron data. The nuclear structure was fixed and the only parameters refined in the 10 K models were the components of the magnetic modes. There are two symmetry-inequivalent Mn^4+^ sites in the asymmetric unit, which share a face in a Mn_2_O_9_ dimer. In our refinements, we have constrained the moments of these sites to be antiparallel. This is justified by the strong AFM direct exchange interactions expected for the half-full t_2g_ orbitals. We tested relaxing this constraint in our final model, but this led to neither a significant improvement in the fitting statistics nor a substantial deviation from the imposed antiparallel configuration. We tested models considering only a single magnetic propagation vector *k* = [0 0 0], as the magnetic intensities can all be indexed on the nuclear cell. At this *k*-point there are 4 irreducible representations (irreps), transforming as *m*Γ_1_^+^, *m*Γ_2_^+^, *m*Γ_1_^–^, and *m*Γ_2_^–^, according to the notation used with ISODISTORT. Illustrations of the spin configurations of each of these modes can be found in the Supporting Information (Figure S2); *m*Γ_1_^+^ has FM components along [010] and AFM components along [100] and [001], *m*Γ_2_^+^ has FM components along [100] and [001] and AFM components along [010], and both *m*Γ_1_^–^ and *m*Γ_2_^–^ have only AFM components along [100], [010], and [001]. The calculated components of the magnetic moments along each direction for each of these modes are included in the Supporting Information in the form of “complete modes details” pages found using ISODISTORT.

**Figure 7:**
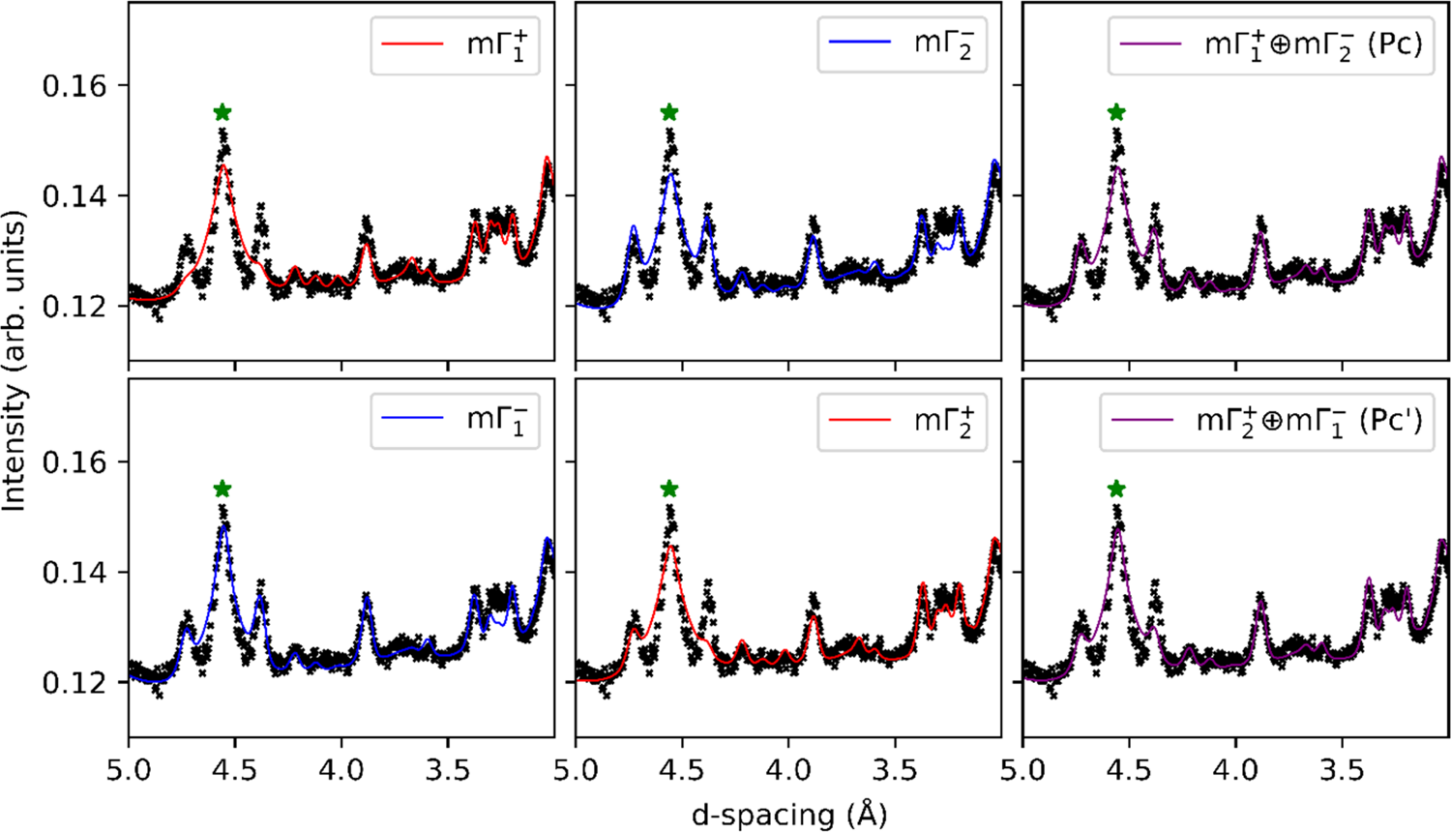
Fits to 10 K powder neutron diffraction data from GEM bank 3 at for Ba_7_Mn_4_O_15_ using single-mode models and dual-mode models, showing the failure of individual modes to capture all magnetic Bragg peak intensity. The underfit intensity in the peak at 4.5 Å is due to the BaCO_3_ impurity, marked with a star symbol.

The results of the models constructed by considering spin orderings that transform as one of these irreps are summarized in Figure 7. The single-irrep models fail to account for all of the magnetic peaks, fitting either the (0 1 2) and (1 0 −2) reflections or the (1 2 −2) and (2 1 −1) reflections, but not both. We therefore performed refinements in which a binary combination of modes was constrained to be active in either the **ac** plane or along **b**, following the symmetry requirements of the unit cell. The three basis vectors spanning each irrep that describe the possible spin orderings correspond with moments aligned along the symmetry-unique direction **b** or in the **ac** plane. We tested all possible combinations exhaustively and report our findings in Table 1. We note that constraining the moments to lie only along **c** made no difference to the fits, despite the moment being unconstrained within the **ac** plane by symmetry. The binary combinations of irreps produce both solutions where only the direction of the moment is modulated (that we will refer to as a spin-wave solution) and solutions where only the magnitude of the moment is modulated (spin density wave), which are highlighted in blue and red boxes, respectively, in Table 1. For a fixed Mn^4+^ valence state, a spin wave is more physical, so we restrict our discussion to these in what follows. However, the assertions about the magnetoelectric ground state that we present below hold true irrespective of this fact.

**Table 1:**
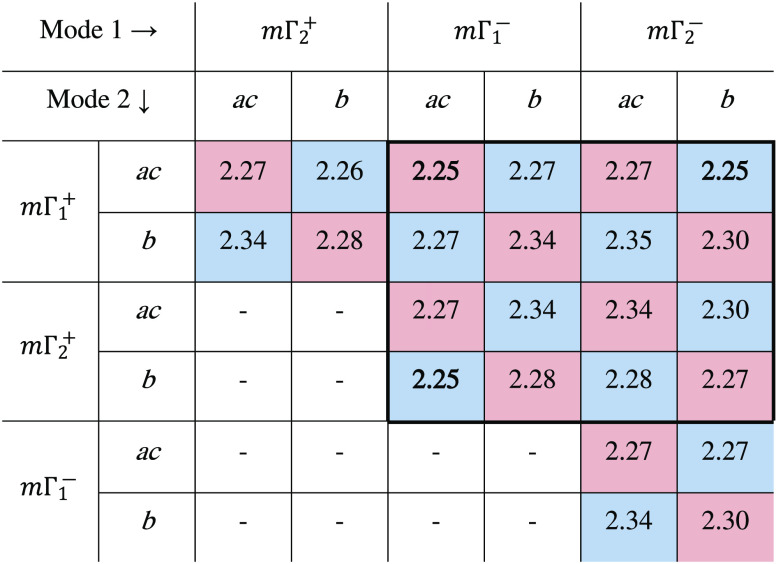
*R*_wp_’s of Refinements of Ba_7_Mn_4_O_15_ Data from GEM Data at 10 K in Which Binary Combinations of Magnetic Modes Were Allowed to Refine^a^

a The boxed region indicates a combination of modes which allow a magnetoelectric (polar) ground state, blue cells indicate spin-wave solutions, and red cells indicate spin density wave solutions.

**Figure 8:**
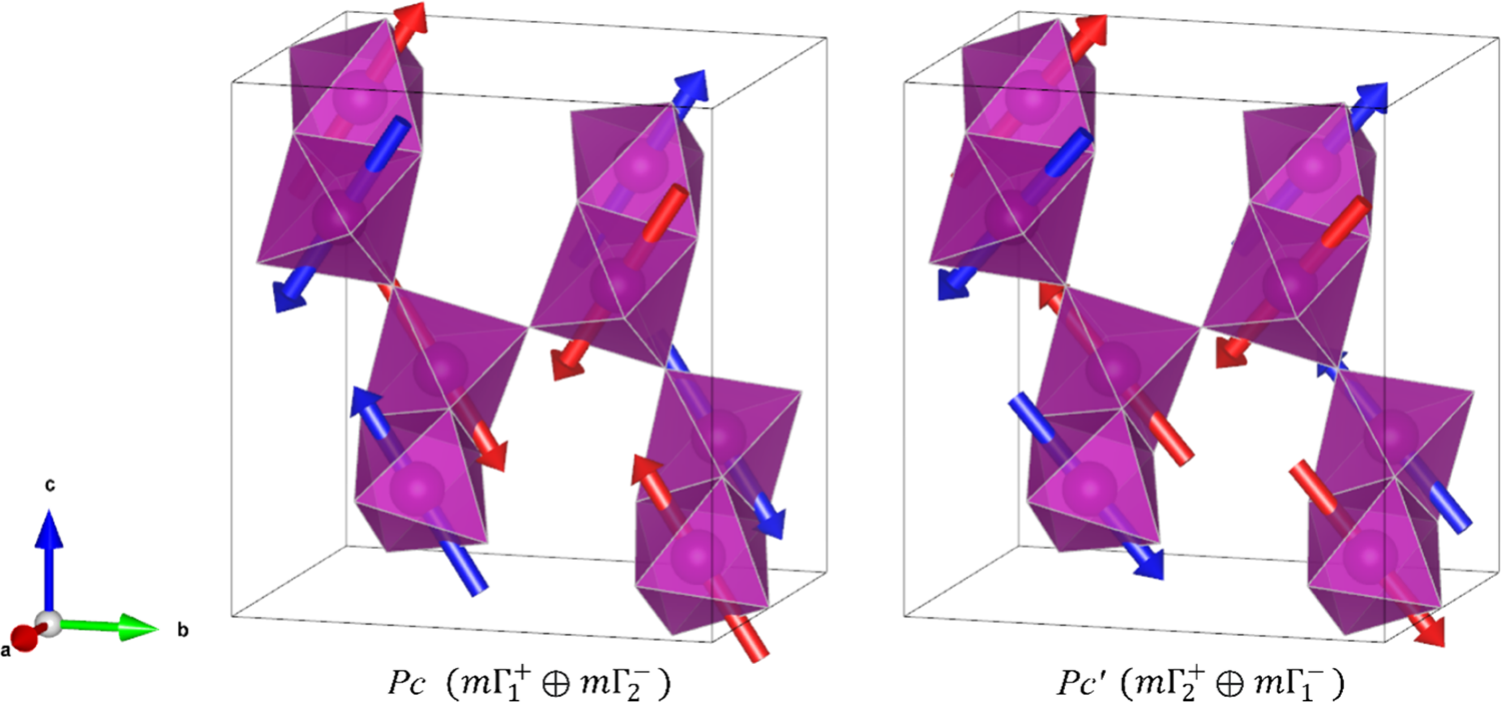
Possible spin configurations for Ba_7_Mn_4_O_15_, where the magnetic moments are constrained to be in the **bc** plane. The total magnetic moment for the *Pc* model is 2.46 μ_B_ (1.6 μ_B_ along **b**, 1.9 μ_B_ along **c**), and the total magnetic moment for the *Pc*′ model is 2.34 μ_B_ (1.5 μ_B_ along **b**, 1.8 μ_B_ along **c**). The two symmetry-unique Mn sites in *P*2_1_/*c* are indicated by blue and red arrows; their coupling is constrained to be AFM as this was found to fit the experimental data best and expected by the strong magnetic direct exchange interactions.

The combinations of magnetic modes which resulted in the best fit to the data are as follows: *m*Γ_1_^+^ with *m*Γ_1_^–^, both along the **c** direction, *m*Γ_1_^+^ along **c** with *m*Γ_2_^–^ along **b**, and *m*Γ_2_^+^ along **b** with *m*Γ_1_^–^ along **c**. These fits are highlighted in bold in Table 1. Refinements in which the irreps were also allowed to refine in **a** were also investigated; we find that constraining the irreps along the **c** and **b** directions does not negatively impact the quality of the fit to the data, in line with literature predictions of the magnetic structure of Sr_7_Mn_4_O_15_.^20,21^ While the components of the magnetic moments were not constrained to be equal along the **b** and **c** lattice directions, they consistently refined to approximately equal values as shown in Figure 8.

Despite demonstrating fitting statistics that equal the best models, we discard the first of the combinations (*m*Γ_1_^+^ with *m*Γ_1_^–^, both parallel to **c**) as it results in a spin density wave with unphysical descriptions of the magnitudes of the moments: namely, the calculated magnitude of the moments on half of the Mn sites are equal to 3.5 μ_B_, while the other half of the sites have magnitudes of 0.1 μ_B_. The remaining combinations of modes result in AFM spin-wave structures with magnetic space groups *Pc* and *Pc*′. The fits to the data from these models are shown in the right-hand-side column of Figure 7, and the spin configurations within the unit cell are shown in Figure 8. We note that the presence of a small BaCO_3_ impurity results in slight underfitting of the peak at 4.5 Å. These two models differ only slightly in the arrangement of the magnetic moments, and both produce magnetic moment magnitudes of around 2.3–2.4 μ_B_. This is slightly reduced from the maximum expected value of 3 μ_B_, which we attribute to the overlap between t_2g_ orbitals of the Mn centers within the Mn_2_O_9_ dimers causing a loss of spin density.

The fact that these two spin configurations are the best-fitting models to our data at low temperature is of significant interest: the joint action of the two magnetic irreps—one of which conserves inversion symmetry (Γ^+^) and one of which violates inversion symmetry (Γ^–^)—results in a structural space group in which inversion symmetry is globally broken.^29^ Indeed, comparing this type of combination (highlighted in Table 1 with a dark box) with combinations where both modes either conserve or do not conserve inversion symmetry, we find that the combinations resulting in a noncentrosymmetric space group fit the data best in all cases. While we are not able to detect an off-centering of any of the high-symmetry positions in Ba_7_Mn_4_O_15_ in our powder diffraction data, the crystallographic analysis indicates that this phase possesses a ground state in which the noncentrosymmetric space group (*Pc*) is a direct result of the magnetic ordering, implying that it may be magnetoelectric and/or multiferroic. A table of the space groups which result from the combination of multiple magnetic modes is shown in the Supporting Information (Table S3).

**Figure 9:**
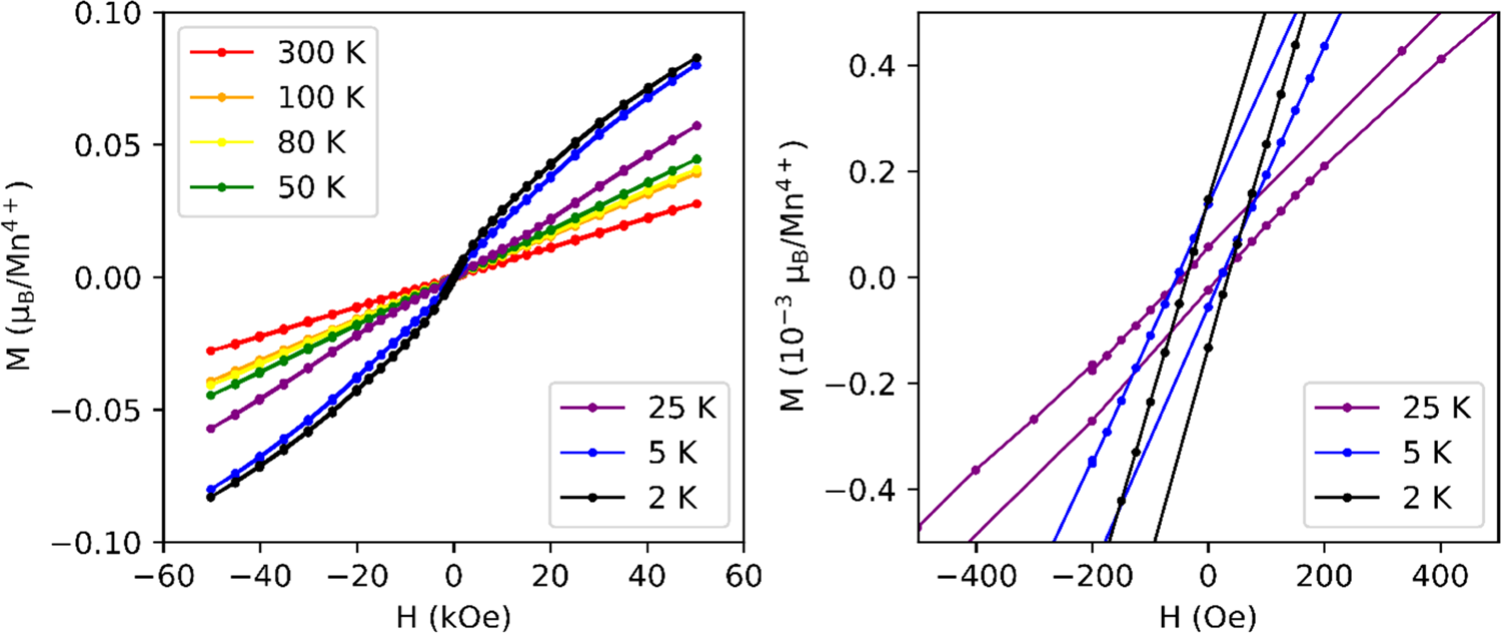
Magnetization versus field results for Ba_7_Mn_4_O_15_.

Figure 9 shows magnetization measurements for Ba_7_Mn_4_O_15_ as a function of the applied field. A small but clear hysteresis is evident at temperatures less than 25 K, with a maximum moment of 0.083 μ_B_ per Mn^4+^ at 2 K and 50 kOe. As this is significantly lower than the effective moment in the paramagnetic region, the ordering is likely to be largely AFM in character, with a small FM component. This can be attributed to a slight canting of the magnetic moments along **b** transforming as the FM irrep *m*Γ_1_^+^, suggesting that there is a small incomplete cancellation of the magnetic moments within the Mn_2_O_9_ dimers along this lattice direction. These magnetization results further support the proposed two-irrep magnetic structure and hence also the proposed polar ground state.

**Figure 10:**
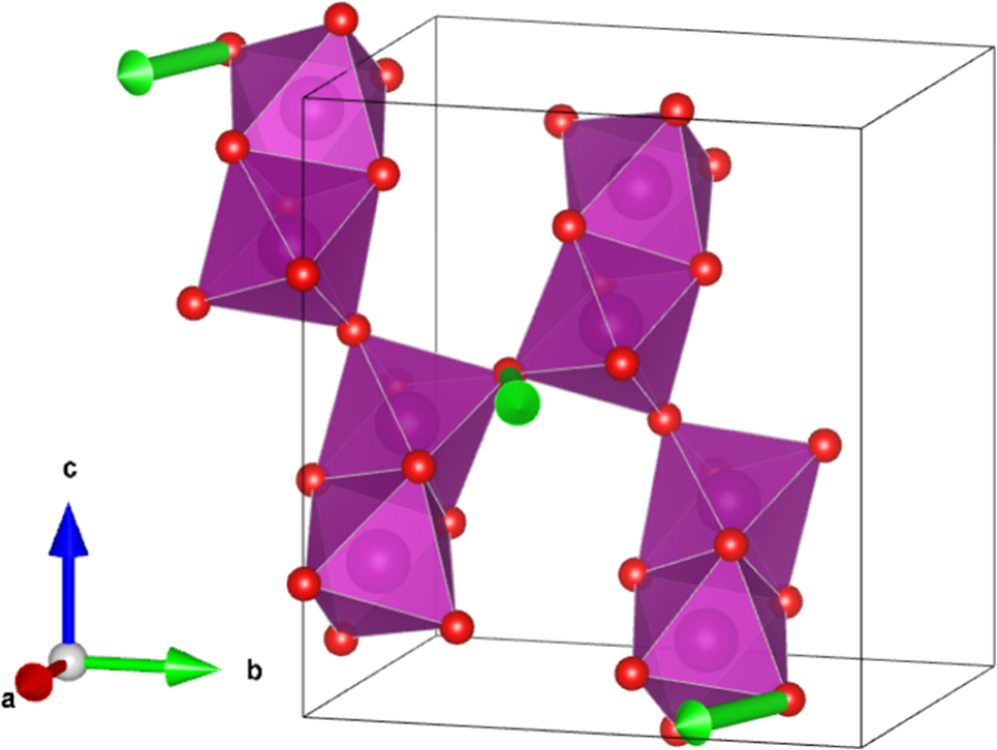
Symmetry-allowed polar displacements (transforming as Γ_2_^–^) of the O6 anion in Ba_7_Mn_4_O_15_ (green polar vectors) in the lower-symmetry *Pc* space group (basis = {(1,0,0),(0,1,0),(0,0,1)}, origin shift = (0,1/4,0) with respect to the *P*2_1_/*c* parent cell).

Figure 10 shows one of the sets of allowed polar displacements of the O6 anion. This site and the Ba3 site are the only high-symmetry positions in the **P**2_1_/**c** unit cell, and thus we chose one of these to demonstrate a possible multiferroic coupling mechanisms since displacements of these atoms from their average positions will always induce a permanent polarization. The polar displacement transforms as the Γ_2_^–^ irrep, which is responsible for the displacive distortions that lead to a symmetry reduction from **P**2_1_/**c** to *Pc* (basis = {(1,0,0),(0,1,0),(0,0,1)}, origin shift = (0,1/4,0) with respect to the parent cell). Notably, polar distortions transforming as this irrep appear irrespective of which magnetic space group (*Pc* or *Pc*′) is assigned, thus our result is invariant to the ambiguity of the precise magnetic structure with respect to these two space groups. Since the noncentrosymmetric ground state would be induced by the magnetic ordering, Ba_7_Mn_4_O_15_ would be classed as a Type II multiferroic. It is important to emphasize that the displacements indicated in Figure 10 are only one of the possible distortion pathways by which magnetoelectric coupling could be realized. Since our proposed distortions are driven by magnetic ordering, the displacement is expected to be on the order of thousandths of an angstrom. Our experiment is not sensitive to displacements of this magnitude in such a complex structure, and so Figure 10 merely shows the symmetry-allowed character of one such possible displacement, and we can infer nothing more about their magnitude or the relative magnitude of other symmetry-allowed atomic displacements. Additionally, it should be noted that the proposed magnetoelectric ground state arises not only as a result of the canting of the magnetic spins, but is due to the combined action of the two magnetic irreps. While a single magnetic irrep solution can lead to canting in this system, it will never result in a multiferroic ground state.

**Figure 11:**
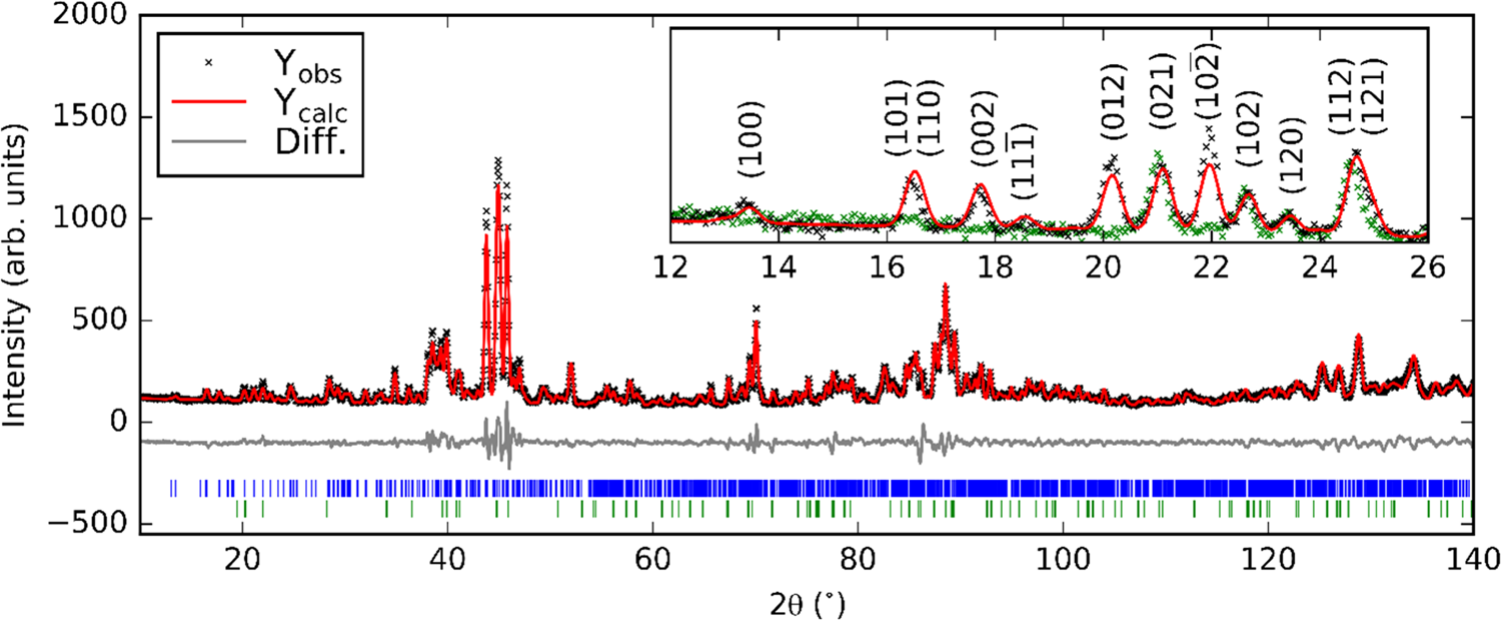
Rietveld refinement against powder neutron diffraction data collected at D2B for Sr_7_Mn_4_O_15_ at 300 K. Blue ticks indicate reflections for the Sr_7_Mn_4_O_15_ phase; green ticks indicate reflections for a small SrMnO_3_ impurity. Inset: magnetic reflections observed at 1.5 K (black crosses) and the same region of 2θ at 300 K (green crosses). The magnetic intensity is well modeled by considering only the *m*Γ_2_^–^ irrep.

**Figure 12:**
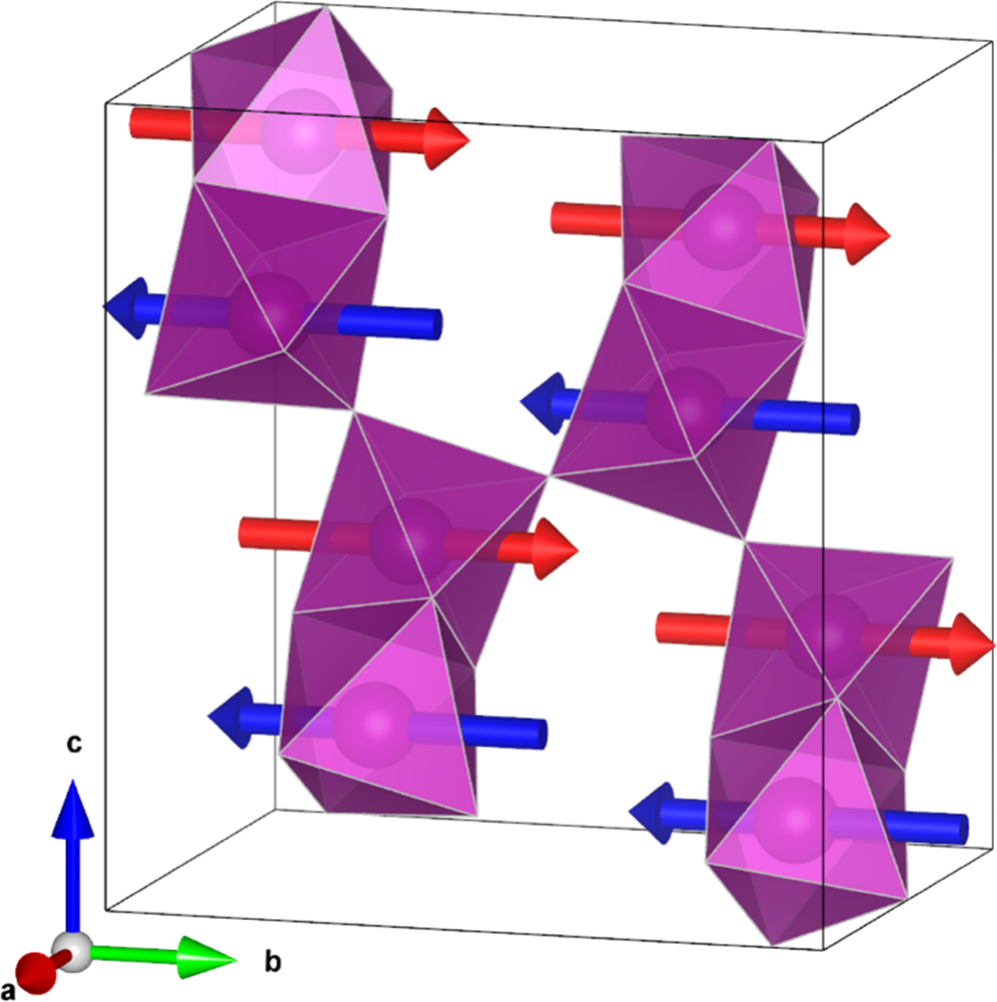
Refined *m*Γ_2_^–^ spin configuration for Sr_7_Mn_4_O_15_ (space group *P*2′_1_/*c*), where the magnetic moments are constrained to be in the **b** direction. The magnetic moment is 2.2 μ_B_. The two symmetry-unique Mn sites in *P*2_1_/*c* are indicated by blue and red arrows; their coupling is constrained to be AFM as this was found to fit the experimental data best and expected by the strong magnetic direct exchange interactions.

Finally, we compare the neutron diffraction results for Ba_7_Mn_4_O_15_ with those of Sr_7_Mn_4_O_15_, for which no direct evidence of a magnetoelectric ground state has previously been reported. A powder neutron diffraction pattern for Sr_7_Mn_4_O_15_ is shown in Figure 11, with the magnetic peaks observed at 1.5 K inset. The most plausible fit to the magnetic peaks is achieved using a model containing only the *m*Γ_2_^–^ irrep along the **b** direction to describe the magnetic moments, consistent with previously published models.^20,21^ The spin configuration refined against the data and transforming as the *m*Γ_2_^–^ irrep is shown in Figure 12. This produces a calculated magnetic moment of 2.2 μ_B_ consistent with the magnitude observed for Ba_7_Mn_4_O_15_, giving us further confidence in our proposed spin-wave solutions. The two-irrep fits necessary to model Ba_7_Mn_4_O_15_ provide no improvement in the quality of the fit to the magnetic data for Sr_7_Mn_4_O_15_, thus the magnetoelectric ground state we report here is specific to Ba_7_Mn_4_O_15_. We view the *Pc* (*m*Γ_1_^+^ ⊕ *m*Γ_2_^–^) structure of Ba_7_Mn_4_O_15_ to be the most likely candidate from our refinements, as the *m*Γ_2_^–^ mode best describes the magnetic ordering in Sr_7_Mn_4_O_15_, and it is to be expected that the exchange pathways have a high degree of similarity in these two materials.

It is difficult to assign the difference in magnetic behaviors between Ba_7_Mn_4_O_15_ and Sr_7_Mn_4_O_15_ to specific structural features due to the low symmetry of the phases. A selection of the nearest-neighbor distances and angles are summarized in Table S3: while the unit cell obviously expands to accommodate the larger Ba^2+^ cations, this expansion does not result in exceptional changes to any of the bond lengths or angles between Mn^4+^ centers. We envisage that substantial future works involving investigating the magnetoelectric ground state of this material, via first-principles calculations, will shed further light on this issue.

## Conclusions

We have successfully synthesized the novel ternary compound Ba_7_Mn_4_O_15_. Powder synchrotron X-ray diffraction analysis indicates that this phase remains in the **P**2_1_/**c** space group in the 100–300 K temperature range. Powder neutron diffraction and SQuID magnetometry indicate that the phase possesses an AFM ground state below 50 K. Careful analysis of this AFM ground state reveals a small FM component and suggests that a pair of magnetic modes, transforming as distinct irreducible representations, act simultaneously, and in doing so couple to a polar distortion. This is the first experimental evidence that this class of materials can support a multiferroic grounds state, and we hope it will stimulate renewed synthetic effort into preparing structural related materials. Further work on Ba_7_Mn_4_O_15_ should focus on direct characterization of the nature of the polar displacement, confirming and demonstrating the switchability of the polar state and performing synthetic work with the aim of enhancing the magnetoelectric ordering temperature.

## Supporting Information Available

Supporting data and figures mentioned in the main text (PDF)
Ba_7_Mn_4_O_15_ from Rietveld refinement of powder synchrotron X-ray diffraction data at 300 K (CIF)
Ba_7_Mn_4_O_15_ from a combined Rietveld refinement of powder synchrotron X-ray diffraction data at 100 K and powder neutron diffraction data at 80 K (CIF)
Components of the magnetic modes derived from ISODISTORT (PDF)
Components of the magnetic modes derived from ISODISTORT (PDF)
Components of the magnetic modes derived from ISODISTORT (PDF)
Components of the magnetic modes derived from ISODISTORT (PDF)

## Supplementary Material

ic2c00889_si_001.pdf

ic2c00889_si_002.cif

ic2c00889_si_003.cif
