# Supplementary material for: Synthesis and Characterization of Magnetoelectric Ba7Mn4O15
Source: Inorg Chem. 2022 Jun 21;61(26):10015–22. doi: 10.1021/acs.inorgchem.2c00889 (PMC9257749; doi:10.1021/acs.inorgchem.2c00889)
Supplement: Supplementary file 1 — ic2c00889_si_001.pdf [file ic2c00889_si_001.pdf]

Supporting information

Synthesis and Characterisation of Magnetoelectric $Ba_7Mn_4O_{15}$

Gabriel R.M. Clarke[a], *Martin R. Lees*[b], *Clemens Ritter*[c], *Ivan da Silva*[d], *Mark S. Senn*[a]*

(*M.Senn@warwick.ac.uk*)

a: Department of Chemistry, University of Warwick, Coventry, CV4 7AL, UK

b: Department of Physics, University of Warwick, Coventry, CV4 7AL, UK

c: Institut Laue-Langevin, 38042 Grenoble Cedex 9, France

d: ISIS Neutron and Muon Facility, Rutherford Appleton Laboratory, Didcot, OX11 0QX, UK

Table S1. Lattice parameters and atomic coordinates refined from powder synchrotron X-ray diffraction data for $Ba_7Mn_4O_{15}$ at 300 K.

Space group	$P2_1/c$				
a (Å)	7.153814(23)				
b (Å)	9.995259(33)				
c (Å)	10.660922(44)				
α (°)	90				
β (°)	92.27597(23)				
γ (°)	90				
Site	x	y	z	Occupancy	B_{ISO} (Å^2)
Ba1	-0.00273(16)	0.18117(12)	0.46748(10)	1	0.261(8)
Ba2	0.34520(17)	0.15387(11)	0.19445(10)	1	0.261(8)
Ba3	0.5	0	0.5	1	0.261(8)
Ba4	0.1740(2)	0.00014(12)	0.83642(13)	1	0.261(8)
Mn1	0.5756(4)	0.1665(3)	0.9233(3)	1	0.47(3)
Mn2	0.7690(4)	0.1688(3)	0.7240(3)	1	0.47(3)
O1	0.5147(1)	0.1027(7)	0.7582(6)	1	0.09(3)
O2	0.6631(11)	0.1867(6)	0.3363(7)	1	0.09(3)
O3	0.3353(11)	0.2418(7)	0.9485(6)	1	0.09(3)
O4	0.8294(1)	0.0839(7)	0.8874(6)	1	0.09(3)
O5	0.6710(12)	0.2369(7)	0.0807(7)	1	0.09(3)
O6	0.5	0	0	1	0.09(3)
O7	0.8337(10)	0.0153(6)	0.6351(6)	1	0.09(3)
O8	-0.0028(12)	0.2483(7)	0.7168(6)	1	0.09(3)

Table S2 Lattice parameters and atomic coordinates refined from combined powder synchrotron X-ray diffraction data and neutron powder diffraction data for $Ba_7Mn_4O_{15}$ at 100 K and 80 K.

Space group	$P2_1/c$				
a (Å)	7.1513(8)				
b (Å)	9.9945(2)				
c (Å)	10.6564(2)				
α (°)	90				
β (°)	92.219(8)				
γ (°)	90				
Site	x	y	z	Occupancy	B_{ISO} (Å^2)
Ba1	-0.00270(16)	0.18109(12)	0.46755(11)	1	0.268(9)
Ba2	0.34522(18)	0.15393(11)	0.19443(10)	1	0.268(9)
Ba3	0.5	0	0.5	1	0.268(9)
Ba4	0.17400(19)	0.00013(12)	0.83646(14)	1	0.268(9)
Mn1	0.5756(4)	0.1665(3)	0.9232(3)	1	0.48(3)
Mn2	0.7689(4)	0.1688(3)	0.7240(3)	1	0.48(3)
O1	0.5146(9)	0.1027(6)	0.7583(6)	1	0.06(3)
O2	0.6626(12)	0.1868(6)	0.3366(7)	1	0.06(3)
O3	0.3355(11)	0.2420(7)	0.9484(6)	1	0.06(3)
O4	0.829(1)	0.0840(7)	0.8873(6)	1	0.06(3)
O5	0.67150(12)	0.2368(7)	0.0807(7)	1	0.06(3)
O6	0.5	0	0	1	0.06(3)
O7	0.834(1)	0.0156(6)	0.6349(6)	1	0.06(3)
O8	-0.0023(12)	0.2480(7)	0.7170(6)	1	0.06(3)

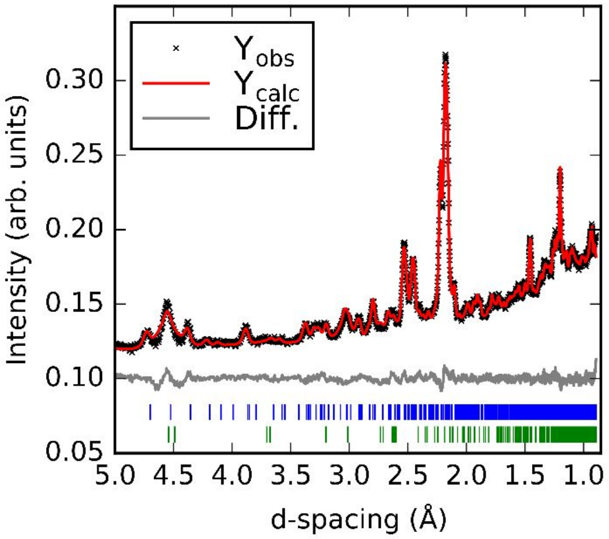

Figure S1. Results of symmetry-adapted Rietveld refinement with Pc magnetic model of GEM data for $Ba_7Mn_4O_{15}$ at 10 K

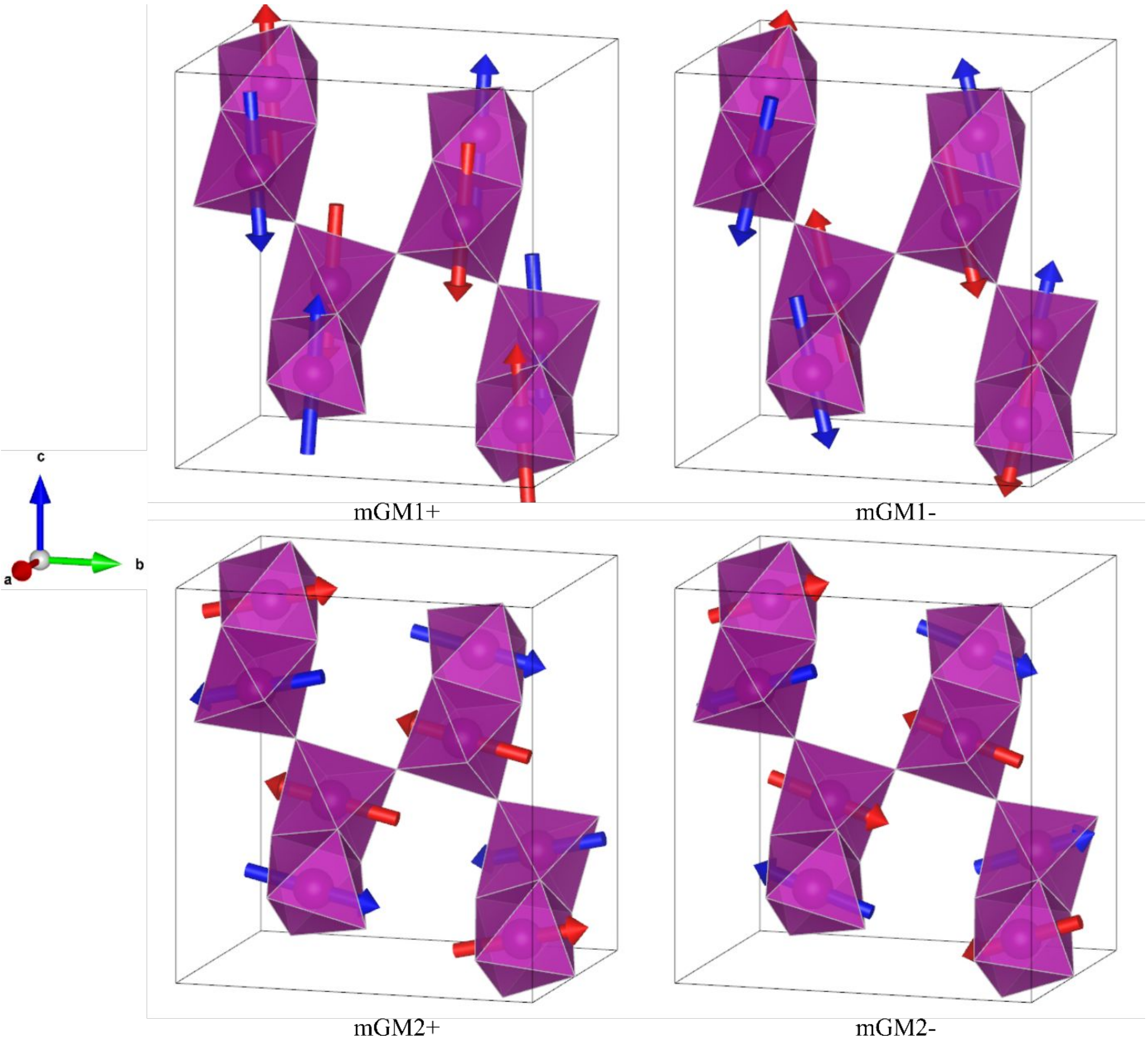

Figure S2. Individual magnetic modes for $Ba_7Mn_4O_{15}$ constrained to the bc-plane in analogy with the best-fitting 2-mode results for $Ba_7Mn_4O_{15}$. $Sr_7Mn_4O_{15}$ is fit best using only $m\Gamma_2^-$, whereas a combination of $m\Gamma^+$ and $m\Gamma^-$ modes produce the best fit for $Ba_7Mn_4O_{15}$.

Table S3. Space groups resulting from different combinations of modes.

	$m\Gamma_1^+$	$m\Gamma_2^+$	$m\Gamma_1^-$	$m\Gamma_2^-$
$m\Gamma_1^+$	$P2_1/c$	$P\bar{1}$	$P2_1$	Pc
$m\Gamma_2^+$	$P\bar{1}$	$P2_1'/c'$	Pc'	$P2_1'$
$m\Gamma_1^-$	$P2_1$	Pc'	$P2_1/c'$	$\overline{P_1'}$
$m\Gamma_2^-$	Pc	$P2_1'$	$\overline{P_1'}$	$P2_1'/c$

Table S4. Selected bond distances and angles for comparison of $Sr_7Mn_4O_{15}$ and $Ba_7Mn_4O_{15}$.

	$Sr_7Mn_4O_{15}$	$Ba_7Mn_4O_{15}$	$Ba_7Mn_4O_{15}$
Temperature	300 K	100 K	10 K

Unit cell volume (Å^3)	680.576(4)	761.698(5)	761.03(3)
Mn1-O6-Mn1 distance (Å)	3.719(2)	3.881(7)	3.897(12)
Mn1-Mn2 intradimer distance (Å)	2.564(2)	2.640(11)	2.638(11)
Mn1-O5-Mn2 distance (Å)	3.697(2)	3.814(5)	3.836(11)
Mn1-O5-Mn2 angle (°)	164.8(2)	171.5(5)	170.9(5)
