# Supplementary material for: Synthesis and Characterization of Magnetoelectric Ba7Mn4O15
Source: Inorg Chem. 2022 Jun 21;61(26):10015–22. doi: 10.1021/acs.inorgchem.2c00889 (PMC9257749; doi:10.1021/acs.inorgchem.2c00889)
Supplement: Supplementary file 6 — ic2c00889_si_006.pdf [file ic2c00889_si_006.pdf]

Parent structure (14 P2_1/c)
a=7.15391, b=9.99955, c=10.66715, alpha=90.00000, beta=92.25612, gamma=90.00000

atom	site	x	y	z	occ
Sr1	4e	-0.00660	0.19242	0.47023	1.00000
Sr2	4e	0.33626	0.15362	0.19824	1.00000
Sr3	2d	0.50000	0.00000	0.50000	1.00000
Sr4	4e	0.17129	-0.01048	-0.15913	1.00000
Mn1	4e	-0.45721	0.17591	-0.08409	1.00000
Mn2	4e	-0.24943	0.16220	-0.27764	1.00000
O1	4e	-0.49545	0.08695	-0.24837	1.00000
O2	4e	-0.34188	0.17164	0.32822	1.00000
O3	4e	0.34196	0.24482	-0.05522	1.00000
O4	4e	-0.16252	0.08810	-0.09972	1.00000
O5	4e	-0.32970	0.24231	0.08607	1.00000
O6	2b	0.50000	0.00000	0.00000	1.00000
O7	4e	-0.16925	0.01053	-0.35861	1.00000
O8	4e	-0.00808	0.24427	-0.28508	1.00000

Subgroup details
14.78 P2_1/c', basis={(1,0,0),(0,1,0),(0,0,1)}, origin=(0,0,0), s=1, i=2, k-active= (0,0,0)

Undistorted superstructure
a=7.15391, b=9.99955, c=10.66715, alpha=90.00000, beta=92.25612, gamma=90.00000

atom	site	x	y	z	occ	displ	mx	my	mz	mag
Sr1_1	4e	0.99340	0.19242	0.47023	1.00000	0.00000	0.00000	0.00000	0.00000	0.00000
Sr2_1	4e	0.33626	0.15362	0.19824	1.00000	0.00000	0.00000	0.00000	0.00000	0.00000
Sr3_1	2d	0.50000	0.00000	0.50000	1.00000	0.00000	0.00000	0.00000	0.00000	0.00000
Sr4_1	4e	0.17129	0.98952	0.84087	1.00000	0.00000	0.00000	0.00000	0.00000	0.00000
Mn1_1	4e	0.54279	0.17591	0.91591	1.00000	0.00000	0.00000	0.00000	0.00000	0.00000
Mn2_1	4e	0.75057	0.16220	0.72236	1.00000	0.00000	0.00000	0.00000	0.00000	0.00000
O1_1	4e	0.50455	0.08695	0.75163	1.00000	0.00000	0.00000	0.00000	0.00000	0.00000
O2_1	4e	0.65812	0.17164	0.32822	1.00000	0.00000	0.00000	0.00000	0.00000	0.00000
O3_1	4e	0.34196	0.24482	0.94478	1.00000	0.00000	0.00000	0.00000	0.00000	0.00000
O4_1	4e	0.83748	0.08810	0.90028	1.00000	0.00000	0.00000	0.00000	0.00000	0.00000
O5_1	4e	0.67030	0.24231	0.08607	1.00000	0.00000	0.00000	0.00000	0.00000	0.00000
O6_1	2b	0.50000	0.00000	0.00000	1.00000	0.00000	0.00000	0.00000	0.00000	0.00000
O7_1	4e	0.83075	0.01053	0.64139	1.00000	0.00000	0.00000	0.00000	0.00000	0.00000
O8_1	4e	0.99192	0.24427	0.71492	1.00000	0.00000	0.00000	0.00000	0.00000	0.00000

Distorted superstructure
a=7.15391, b=9.99955, c=10.66715, alpha=90.00000, beta=92.25612, gamma=90.00000

atom	site	x	y	z	occ	displ	mx	my	mz	mag
Sr1_1	4e	0.99340	0.19242	0.47023	1.00000	0.00000	0.00000	0.00000	0.00000	0.00000
Sr2_1	4e	0.33626	0.15362	0.19824	1.00000	0.00000	0.00000	0.00000	0.00000	0.00000
Sr3_1	2d	0.50000	0.00000	0.50000	1.00000	0.00000	0.00000	0.00000	0.00000	0.00000
Sr4_1	4e	0.17129	0.98952	0.84087	1.00000	0.00000	0.00000	0.00000	0.00000	0.00000
Mn1_1	4e	0.54279	0.17591	0.91591	1.00000	0.00000	0.00000	0.00000	0.00000	0.00000
Mn2_1	4e	0.75057	0.16220	0.72236	1.00000	0.00000	0.00000	0.00000	0.00000	0.00000
O1_1	4e	0.50455	0.08695	0.75163	1.00000	0.00000	0.00000	0.00000	0.00000	0.00000
O2_1	4e	0.65812	0.17164	0.32822	1.00000	0.00000	0.00000	0.00000	0.00000	0.00000
O3_1	4e	0.34196	0.24482	0.94478	1.00000	0.00000	0.00000	0.00000	0.00000	0.00000
O4_1	4e	0.83748	0.08810	0.90028	1.00000	0.00000	0.00000	0.00000	0.00000	0.00000
O5_1	4e	0.67030	0.24231	0.08607	1.00000	0.00000	0.00000	0.00000	0.00000	0.00000
O6_1	2b	0.50000	0.00000	0.00000	1.00000	0.00000	0.00000	0.00000	0.00000	0.00000
O7_1	4e	0.83075	0.01053	0.64139	1.00000	0.00000	0.00000	0.00000	0.00000	0.00000
O8_1	4e	0.99192	0.24427	0.71492	1.00000	0.00000	0.00000	0.00000	0.00000	0.00000

Displacive mode definitions

atom	x	y	z	dx	dy	dz
P2_1/c[0,0,0]GM1+(a)[Sr1:e:dsp]A_1(a) normfactor = 0.04687						
Sr1_1	0.99340	0.19242	0.47023	0.0000	0.0000	1.0000
	0.00660	0.69242	0.02977	0.0000	0.0000	-1.0000
	0.00660	0.80758	0.52977	0.0000	0.0000	-1.0000
	0.99340	0.30758	0.97023	0.0000	0.0000	1.0000
P2_1/c[0,0,0]GM1+(a)[Sr1:e:dsp]A_2(a) normfactor = 0.06995						
Sr1_1	0.99340	0.19242	0.47023	1.0000	0.0000	0.0264
	0.00660	0.69242	0.02977	-1.0000	0.0000	-0.0264
	0.00660	0.80758	0.52977	-1.0000	0.0000	-0.0264
	0.99340	0.30758	0.97023	1.0000	0.0000	0.0264
P2_1/c[0,0,0]GM1+(a)[Sr1:e:dsp]A_3(a) normfactor = 0.05000						
Sr1_1	0.99340	0.19242	0.47023	0.0000	1.0000	0.0000
	0.00660	0.69242	0.02977	0.0000	1.0000	0.0000
	0.00660	0.80758	0.52977	0.0000	-1.0000	0.0000
	0.99340	0.30758	0.97023	0.0000	-1.0000	0.0000
P2_1/c[0,0,0]GM1+(a)[Sr2:e:dsp]A_1(a) normfactor = 0.04687						
Sr2_1	0.33626	0.15362	0.19824	0.0000	0.0000	1.0000
	0.66374	0.65362	0.30176	0.0000	0.0000	-1.0000
	0.66374	0.84638	0.80176	0.0000	0.0000	-1.0000
	0.33626	0.34638	0.69824	0.0000	0.0000	1.0000
P2_1/c[0,0,0]GM1+(a)[Sr2:e:dsp]A_2(a) normfactor = 0.06995						
Sr2_1	0.33626	0.15362	0.19824	1.0000	0.0000	0.0264
	0.66374	0.65362	0.30176	-1.0000	0.0000	-0.0264

```
       0.66374  0.84638  0.80176 -1.0000  0.0000 -0.0264
       0.33626  0.34638  0.69824  1.0000  0.0000  0.0264

P2_1/c[0,0,0]GM1+(a)[Sr2:e:dsp]A_3(a) normfactor = 0.05000
Sr2_1  0.33626  0.15362  0.19824  0.0000  1.0000  0.0000
       0.66374  0.65362  0.30176  0.0000  1.0000  0.0000
       0.66374  0.84638  0.80176  0.0000 -1.0000  0.0000
       0.33626  0.34638  0.69824  0.0000 -1.0000  0.0000

P2_1/c[0,0,0]GM1+(a)[Sr4:e:dsp]A_1(a) normfactor = 0.04687
Sr4_1  0.17129  0.98952  0.84087  0.0000  0.0000  1.0000
       0.82871  0.48952  0.65913  0.0000  0.0000 -1.0000
       0.82871  0.01048  0.15913  0.0000  0.0000 -1.0000
       0.17129  0.51048  0.34087  0.0000  0.0000  1.0000

P2_1/c[0,0,0]GM1+(a)[Sr4:e:dsp]A_2(a) normfactor = 0.06995
Sr4_1  0.17129  0.98952  0.84087  1.0000  0.0000  0.0264
       0.82871  0.48952  0.65913 -1.0000  0.0000 -0.0264
       0.82871  0.01048  0.15913 -1.0000  0.0000 -0.0264
       0.17129  0.51048  0.34087  1.0000  0.0000  0.0264

P2_1/c[0,0,0]GM1+(a)[Sr4:e:dsp]A_3(a) normfactor = 0.05000
Sr4_1  0.17129  0.98952  0.84087  0.0000  1.0000  0.0000
       0.82871  0.48952  0.65913  0.0000  1.0000  0.0000
       0.82871  0.01048  0.15913  0.0000 -1.0000  0.0000
       0.17129  0.51048  0.34087  0.0000 -1.0000  0.0000

P2_1/c[0,0,0]GM1+(a)[Mn1:e:dsp]A_1(a) normfactor = 0.04687
Mn1_1  0.54279  0.17591  0.91591  0.0000  0.0000  1.0000
       0.45721  0.67591  0.58409  0.0000  0.0000 -1.0000
       0.45721  0.82409  0.08409  0.0000  0.0000 -1.0000
       0.54279  0.32409  0.41591  0.0000  0.0000  1.0000

P2_1/c[0,0,0]GM1+(a)[Mn1:e:dsp]A_2(a) normfactor = 0.06995
Mn1_1  0.54279  0.17591  0.91591  1.0000  0.0000  0.0264
       0.45721  0.67591  0.58409 -1.0000  0.0000 -0.0264
       0.45721  0.82409  0.08409 -1.0000  0.0000 -0.0264
       0.54279  0.32409  0.41591  1.0000  0.0000  0.0264

P2_1/c[0,0,0]GM1+(a)[Mn1:e:dsp]A_3(a) normfactor = 0.05000
Mn1_1  0.54279  0.17591  0.91591  0.0000  1.0000  0.0000
       0.45721  0.67591  0.58409  0.0000  1.0000  0.0000
       0.45721  0.82409  0.08409  0.0000 -1.0000  0.0000
       0.54279  0.32409  0.41591  0.0000 -1.0000  0.0000

P2_1/c[0,0,0]GM1+(a)[Mn2:e:dsp]A_1(a) normfactor = 0.04687
Mn2_1  0.75057  0.16220  0.72236  0.0000  0.0000  1.0000
       0.24943  0.66220  0.77764  0.0000  0.0000 -1.0000
       0.24943  0.83780  0.27764  0.0000  0.0000 -1.0000
       0.75057  0.33780  0.22236  0.0000  0.0000  1.0000

P2_1/c[0,0,0]GM1+(a)[Mn2:e:dsp]A_2(a) normfactor = 0.06995
Mn2_1  0.75057  0.16220  0.72236  1.0000  0.0000  0.0264
       0.24943  0.66220  0.77764 -1.0000  0.0000 -0.0264
       0.24943  0.83780  0.27764 -1.0000  0.0000 -0.0264
       0.75057  0.33780  0.22236  1.0000  0.0000  0.0264

P2_1/c[0,0,0]GM1+(a)[Mn2:e:dsp]A_3(a) normfactor = 0.05000
Mn2_1  0.75057  0.16220  0.72236  0.0000  1.0000  0.0000
       0.24943  0.66220  0.77764  0.0000  1.0000  0.0000
       0.24943  0.83780  0.27764  0.0000 -1.0000  0.0000
```

```
       0.75057  0.33780  0.22236  0.0000 -1.0000  0.0000

P2_1/c[0,0,0]GM1+(a)[O1:e:dsp]A_1(a) normfactor = 0.04687
O1_1   0.50455  0.08695  0.75163  0.0000  0.0000  1.0000
       0.49545  0.58695  0.74837  0.0000  0.0000 -1.0000
       0.49545  0.91305  0.24837  0.0000  0.0000 -1.0000
       0.50455  0.41305  0.25163  0.0000  0.0000  1.0000

P2_1/c[0,0,0]GM1+(a)[O1:e:dsp]A_2(a) normfactor = 0.06995
O1_1   0.50455  0.08695  0.75163  1.0000  0.0000  0.0264
       0.49545  0.58695  0.74837 -1.0000  0.0000 -0.0264
       0.49545  0.91305  0.24837 -1.0000  0.0000 -0.0264
       0.50455  0.41305  0.25163  1.0000  0.0000  0.0264

P2_1/c[0,0,0]GM1+(a)[O1:e:dsp]A_3(a) normfactor = 0.05000
O1_1   0.50455  0.08695  0.75163  0.0000  1.0000  0.0000
       0.49545  0.58695  0.74837  0.0000  1.0000  0.0000
       0.49545  0.91305  0.24837  0.0000 -1.0000  0.0000
       0.50455  0.41305  0.25163  0.0000 -1.0000  0.0000

P2_1/c[0,0,0]GM1+(a)[O2:e:dsp]A_1(a) normfactor = 0.04687
O2_1   0.65812  0.17164  0.32822  0.0000  0.0000  1.0000
       0.34188  0.67164  0.17178  0.0000  0.0000 -1.0000
       0.34188  0.82836  0.67178  0.0000  0.0000 -1.0000
       0.65812  0.32836  0.82822  0.0000  0.0000  1.0000

P2_1/c[0,0,0]GM1+(a)[O2:e:dsp]A_2(a) normfactor = 0.06995
O2_1   0.65812  0.17164  0.32822  1.0000  0.0000  0.0264
       0.34188  0.67164  0.17178 -1.0000  0.0000 -0.0264
       0.34188  0.82836  0.67178 -1.0000  0.0000 -0.0264
       0.65812  0.32836  0.82822  1.0000  0.0000  0.0264

P2_1/c[0,0,0]GM1+(a)[O2:e:dsp]A_3(a) normfactor = 0.05000
O2_1   0.65812  0.17164  0.32822  0.0000  1.0000  0.0000
       0.34188  0.67164  0.17178  0.0000  1.0000  0.0000
       0.34188  0.82836  0.67178  0.0000 -1.0000  0.0000
       0.65812  0.32836  0.82822  0.0000 -1.0000  0.0000

P2_1/c[0,0,0]GM1+(a)[O3:e:dsp]A_1(a) normfactor = 0.04687
O3_1   0.34196  0.24482  0.94478  0.0000  0.0000  1.0000
       0.65804  0.74482  0.55522  0.0000  0.0000 -1.0000
       0.65804  0.75518  0.05522  0.0000  0.0000 -1.0000
       0.34196  0.25518  0.44478  0.0000  0.0000  1.0000

P2_1/c[0,0,0]GM1+(a)[O3:e:dsp]A_2(a) normfactor = 0.06995
O3_1   0.34196  0.24482  0.94478  1.0000  0.0000  0.0264
       0.65804  0.74482  0.55522 -1.0000  0.0000 -0.0264
       0.65804  0.75518  0.05522 -1.0000  0.0000 -0.0264
       0.34196  0.25518  0.44478  1.0000  0.0000  0.0264

P2_1/c[0,0,0]GM1+(a)[O3:e:dsp]A_3(a) normfactor = 0.05000
O3_1   0.34196  0.24482  0.94478  0.0000  1.0000  0.0000
       0.65804  0.74482  0.55522  0.0000  1.0000  0.0000
       0.65804  0.75518  0.05522  0.0000 -1.0000  0.0000
       0.34196  0.25518  0.44478  0.0000 -1.0000  0.0000

P2_1/c[0,0,0]GM1+(a)[O4:e:dsp]A_1(a) normfactor = 0.04687
O4_1   0.83748  0.08810  0.90028  0.0000  0.0000  1.0000
       0.16252  0.58810  0.59972  0.0000  0.0000 -1.0000
       0.16252  0.91190  0.09972  0.0000  0.0000 -1.0000
       0.83748  0.41190  0.40028  0.0000  0.0000  1.0000
```

```
P2_1/c[0,0,0]GM1+(a)[O4:e:dsp]A_2(a) normfactor = 0.06995
O4_1   0.83748  0.08810  0.90028  1.0000  0.0000  0.0264
       0.16252  0.58810  0.59972 -1.0000  0.0000 -0.0264
       0.16252  0.91190  0.09972 -1.0000  0.0000 -0.0264
       0.83748  0.41190  0.40028  1.0000  0.0000  0.0264

P2_1/c[0,0,0]GM1+(a)[O4:e:dsp]A_3(a) normfactor = 0.05000
O4_1   0.83748  0.08810  0.90028  0.0000  1.0000  0.0000
       0.16252  0.58810  0.59972  0.0000  1.0000  0.0000
       0.16252  0.91190  0.09972  0.0000 -1.0000  0.0000
       0.83748  0.41190  0.40028  0.0000 -1.0000  0.0000

P2_1/c[0,0,0]GM1+(a)[O5:e:dsp]A_1(a) normfactor = 0.04687
O5_1   0.67030  0.24231  0.08607  0.0000  0.0000  1.0000
       0.32970  0.74231  0.41393  0.0000  0.0000 -1.0000
       0.32970  0.75769  0.91393  0.0000  0.0000 -1.0000
       0.67030  0.25769  0.58607  0.0000  0.0000  1.0000

P2_1/c[0,0,0]GM1+(a)[O5:e:dsp]A_2(a) normfactor = 0.06995
O5_1   0.67030  0.24231  0.08607  1.0000  0.0000  0.0264
       0.32970  0.74231  0.41393 -1.0000  0.0000 -0.0264
       0.32970  0.75769  0.91393 -1.0000  0.0000 -0.0264
       0.67030  0.25769  0.58607  1.0000  0.0000  0.0264

P2_1/c[0,0,0]GM1+(a)[O5:e:dsp]A_3(a) normfactor = 0.05000
O5_1   0.67030  0.24231  0.08607  0.0000  1.0000  0.0000
       0.32970  0.74231  0.41393  0.0000  1.0000  0.0000
       0.32970  0.75769  0.91393  0.0000 -1.0000  0.0000
       0.67030  0.25769  0.58607  0.0000 -1.0000  0.0000

P2_1/c[0,0,0]GM1+(a)[O7:e:dsp]A_1(a) normfactor = 0.04687
O7_1   0.83075  0.01053  0.64139  0.0000  0.0000  1.0000
       0.16925  0.51053  0.85861  0.0000  0.0000 -1.0000
       0.16925  0.98947  0.35861  0.0000  0.0000 -1.0000
       0.83075  0.48947  0.14139  0.0000  0.0000  1.0000

P2_1/c[0,0,0]GM1+(a)[O7:e:dsp]A_2(a) normfactor = 0.06995
O7_1   0.83075  0.01053  0.64139  1.0000  0.0000  0.0264
       0.16925  0.51053  0.85861 -1.0000  0.0000 -0.0264
       0.16925  0.98947  0.35861 -1.0000  0.0000 -0.0264
       0.83075  0.48947  0.14139  1.0000  0.0000  0.0264

P2_1/c[0,0,0]GM1+(a)[O7:e:dsp]A_3(a) normfactor = 0.05000
O7_1   0.83075  0.01053  0.64139  0.0000  1.0000  0.0000
       0.16925  0.51053  0.85861  0.0000  1.0000  0.0000
       0.16925  0.98947  0.35861  0.0000 -1.0000  0.0000
       0.83075  0.48947  0.14139  0.0000 -1.0000  0.0000

P2_1/c[0,0,0]GM1+(a)[O8:e:dsp]A_1(a) normfactor = 0.04687
O8_1   0.99192  0.24427  0.71492  0.0000  0.0000  1.0000
       0.00808  0.74427  0.78508  0.0000  0.0000 -1.0000
       0.00808  0.75573  0.28508  0.0000  0.0000 -1.0000
       0.99192  0.25573  0.21492  0.0000  0.0000  1.0000

P2_1/c[0,0,0]GM1+(a)[O8:e:dsp]A_2(a) normfactor = 0.06995
O8_1   0.99192  0.24427  0.71492  1.0000  0.0000  0.0264
       0.00808  0.74427  0.78508 -1.0000  0.0000 -0.0264
       0.00808  0.75573  0.28508 -1.0000  0.0000 -0.0264
       0.99192  0.25573  0.21492  1.0000  0.0000  0.0264
```

P2_1/c[0,0,0]GM1+(a)[O8:e:dsp]A_3(a) normfactor = 0.05000

O8_1	0.99192	0.24427	0.71492	0.0000	1.0000	0.0000
	0.00808	0.74427	0.78508	0.0000	1.0000	0.0000
	0.00808	0.75573	0.28508	0.0000	-1.0000	0.0000
	0.99192	0.25573	0.21492	0.0000	-1.0000	0.0000

Displacive mode amplitudes

**mode**	**As**	**Ap**	**dmax**
[0,0,0]GM1+[Sr1:e:dsp]A_1(a)	0.00000	0.00000	0.00000
[0,0,0]GM1+[Sr1:e:dsp]A_2(a)	0.00000	0.00000	0.00000
[0,0,0]GM1+[Sr1:e:dsp]A_3(a)	0.00000	0.00000	0.00000
[0,0,0]GM1+[Sr2:e:dsp]A_1(a)	0.00000	0.00000	0.00000
[0,0,0]GM1+[Sr2:e:dsp]A_2(a)	0.00000	0.00000	0.00000
[0,0,0]GM1+[Sr2:e:dsp]A_3(a)	0.00000	0.00000	0.00000
[0,0,0]GM1+[Sr4:e:dsp]A_1(a)	0.00000	0.00000	0.00000
[0,0,0]GM1+[Sr4:e:dsp]A_2(a)	0.00000	0.00000	0.00000
[0,0,0]GM1+[Sr4:e:dsp]A_3(a)	0.00000	0.00000	0.00000
[0,0,0]GM1+[Mn1:e:dsp]A_1(a)	0.00000	0.00000	0.00000
[0,0,0]GM1+[Mn1:e:dsp]A_2(a)	0.00000	0.00000	0.00000
[0,0,0]GM1+[Mn1:e:dsp]A_3(a)	0.00000	0.00000	0.00000
[0,0,0]GM1+[Mn2:e:dsp]A_1(a)	0.00000	0.00000	0.00000
[0,0,0]GM1+[Mn2:e:dsp]A_2(a)	0.00000	0.00000	0.00000
[0,0,0]GM1+[Mn2:e:dsp]A_3(a)	0.00000	0.00000	0.00000
[0,0,0]GM1+[O1:e:dsp]A_1(a)	0.00000	0.00000	0.00000
[0,0,0]GM1+[O1:e:dsp]A_2(a)	0.00000	0.00000	0.00000
[0,0,0]GM1+[O1:e:dsp]A_3(a)	0.00000	0.00000	0.00000
[0,0,0]GM1+[O2:e:dsp]A_1(a)	0.00000	0.00000	0.00000
[0,0,0]GM1+[O2:e:dsp]A_2(a)	0.00000	0.00000	0.00000
[0,0,0]GM1+[O2:e:dsp]A_3(a)	0.00000	0.00000	0.00000
[0,0,0]GM1+[O3:e:dsp]A_1(a)	0.00000	0.00000	0.00000
[0,0,0]GM1+[O3:e:dsp]A_2(a)	0.00000	0.00000	0.00000
[0,0,0]GM1+[O3:e:dsp]A_3(a)	0.00000	0.00000	0.00000
[0,0,0]GM1+[O4:e:dsp]A_1(a)	0.00000	0.00000	0.00000
[0,0,0]GM1+[O4:e:dsp]A_2(a)	0.00000	0.00000	0.00000
[0,0,0]GM1+[O4:e:dsp]A_3(a)	0.00000	0.00000	0.00000
[0,0,0]GM1+[O5:e:dsp]A_1(a)	0.00000	0.00000	0.00000
[0,0,0]GM1+[O5:e:dsp]A_2(a)	0.00000	0.00000	0.00000
[0,0,0]GM1+[O5:e:dsp]A_3(a)	0.00000	0.00000	0.00000
[0,0,0]GM1+[O7:e:dsp]A_1(a)	0.00000	0.00000	0.00000
[0,0,0]GM1+[O7:e:dsp]A_2(a)	0.00000	0.00000	0.00000
[0,0,0]GM1+[O7:e:dsp]A_3(a)	0.00000	0.00000	0.00000
[0,0,0]GM1+[O8:e:dsp]A_1(a)	0.00000	0.00000	0.00000
[0,0,0]GM1+[O8:e:dsp]A_2(a)	0.00000	0.00000	0.00000
[0,0,0]GM1+[O8:e:dsp]A_3(a)	0.00000	0.00000	0.00000
[0,0,0]GM1+ all	0.00000	0.00000	
Overall	0.00000	0.00000	

Magnetic mode definitions

**atom**	**x**	**y**	**z**	**dmx**	**dmy**	**dmz**
P2_1/c[0,0,0]mGM1-(a)[Mn1:e:mag]A_1(a) normfactor = 0.04687						
Mn1_1	0.54279	0.17591	0.91591	0.0000	0.0000	1.0000
	0.45721	0.67591	0.58409	0.0000	0.0000	-1.0000
	0.45721	0.82409	0.08409	0.0000	0.0000	-1.0000
	0.54279	0.32409	0.41591	0.0000	0.0000	1.0000
P2_1/c[0,0,0]mGM1-(a)[Mn1:e:mag]A_2(a) normfactor = 0.06995						
Mn1_1	0.54279	0.17591	0.91591	1.0000	0.0000	0.0264
	0.45721	0.67591	0.58409	-1.0000	0.0000	-0.0264

```
       0.45721  0.82409  0.08409 -1.0000  0.0000 -0.0264
       0.54279  0.32409  0.41591  1.0000  0.0000  0.0264

P2_1/c[0,0,0]mGM1-(a)[Mn1:e:mag]A_3(a) normfactor = 0.05000
Mn1_1  0.54279  0.17591  0.91591  0.0000  1.0000  0.0000
       0.45721  0.67591  0.58409  0.0000  1.0000  0.0000
       0.45721  0.82409  0.08409  0.0000 -1.0000  0.0000
       0.54279  0.32409  0.41591  0.0000 -1.0000  0.0000

P2_1/c[0,0,0]mGM1-(a)[Mn2:e:mag]A_1(a) normfactor = 0.04687
Mn2_1  0.75057  0.16220  0.72236  0.0000  0.0000  1.0000
       0.24943  0.66220  0.77764  0.0000  0.0000 -1.0000
       0.24943  0.83780  0.27764  0.0000  0.0000 -1.0000
       0.75057  0.33780  0.22236  0.0000  0.0000  1.0000

P2_1/c[0,0,0]mGM1-(a)[Mn2:e:mag]A_2(a) normfactor = 0.06995
Mn2_1  0.75057  0.16220  0.72236  1.0000  0.0000  0.0264
       0.24943  0.66220  0.77764 -1.0000  0.0000 -0.0264
       0.24943  0.83780  0.27764 -1.0000  0.0000 -0.0264
       0.75057  0.33780  0.22236  1.0000  0.0000  0.0264

P2_1/c[0,0,0]mGM1-(a)[Mn2:e:mag]A_3(a) normfactor = 0.05000
Mn2_1  0.75057  0.16220  0.72236  0.0000  1.0000  0.0000
       0.24943  0.66220  0.77764  0.0000  1.0000  0.0000
       0.24943  0.83780  0.27764  0.0000 -1.0000  0.0000
       0.75057  0.33780  0.22236  0.0000 -1.0000  0.0000
```

Magnetic mode amplitudes

mode	As	Ap	mmax
[0,0,0]mGM1-[Mn1:e:mag]A_1(a)	0.00000	0.00000	0.00000
[0,0,0]mGM1-[Mn1:e:mag]A_2(a)	0.00000	0.00000	0.00000
[0,0,0]mGM1-[Mn1:e:mag]A_3(a)	0.00000	0.00000	0.00000
[0,0,0]mGM1-[Mn2:e:mag]A_1(a)	0.00000	0.00000	0.00000
[0,0,0]mGM1-[Mn2:e:mag]A_2(a)	0.00000	0.00000	0.00000
[0,0,0]mGM1-[Mn2:e:mag]A_3(a)	0.00000	0.00000	0.00000
[0,0,0]mGM1- all	0.00000	0.00000	
Overall	0.00000	0.00000	

Parent-cell strain mode definitions

```
    e1      e2      e3      e4      e5      e6
P2_1/c[0,0,0]GM1+(a)strain_1(a) normfactor = 1.00000
  1.0000  0.0000  0.0000  0.0000  0.0000  0.0000
P2_1/c[0,0,0]GM1+(a)strain_2(a) normfactor = 1.41421
  0.0000  0.0000  0.0000  0.0000  1.0000  0.0000
P2_1/c[0,0,0]GM1+(a)strain_3(a) normfactor = 1.00000
  0.0000  1.0000  0.0000  0.0000  0.0000  0.0000
P2_1/c[0,0,0]GM1+(a)strain_4(a) normfactor = 1.00000
  0.0000  0.0000  1.0000  0.0000  0.0000  0.0000
```

Parent-cell strain mode amplitudes

mode	amplitude
[0,0,0]GM1+strain_1(a)	0.00000
[0,0,0]GM1+strain_2(a)	0.00000
[0,0,0]GM1+strain_3(a)	0.00000
[0,0,0]GM1+strain_4(a)	0.00000
